# Brazilian kefir fraction mitigates the Alzheimer-like phenotype in *Drosophila melanogaster* with β-amyloid overexpression model

**DOI:** 10.1038/s41598-024-76601-9

**Published:** 2024-10-26

**Authors:** Serena Mares Malta, Tamiris Sabrina Rodrigues, Matheus Henrique Silva, Alexandre Souza Marquez, Rafael Bernardes Ferreira, Fernanda Naves Araújo do Prado Mascarenhas, Renata Graciele Zanon, Lucas Matos Martins Bernardes, Letícia Leandro Batista, Murillo Néia Thomaz da Silva, Débora de Oliveira Santos, Ana Carolina Costa Santos, Ana Paula Mendes-Silva, Foued Salmen Spindola, Carlos Ueira-Vieira

**Affiliations:** 1 Laboratory of Genetics, Institute of Biotechnology, Federal University of Uberlândia, Acre Street, 2E building, room 230, Uberlândia, MG 38405-319 Brazil; 2 Institute of Biomedical Sciences, Federal University of Uberlândia, Uberlândia, MG Brazil; 3 Institute of Developmental Biology and Neurobiology, Johannes Gutenberg University Mainz, Mainz, Germany; 4 Institute of Chemistry, Federal University of Uberlândia, Uberlândia, MG Brazil; 5 Faculty of Odontology, Federal University of Uberlândia, Uberlândia, MG Brazil; 6 Department of Psychiatry, University of Saskatchewan, Saskatoon, SK Canada

**Keywords:** Alzheimer’s disease, *Drosophila melanogaster*, Kefir fractions, Peptides, Biotechnology, Genetics

## Abstract

Alzheimer’s disease (AD) is a progressive neurodegenerative condition and the primary form of dementia among elderly people. The amyloidogenic hypothesis is the main theory that explains this phenomenon and describes the extracellular accumulation of amyloid beta (Aβ) peptides. Model organisms such as *Drosophila melanogaster* have been utilized to improve the understanding of this disease and its treatment. This study evaluated the effects of peptide and metabolic fractions of Brazilian kefir on a strain of *D. melanogaster* that expresses human Aβ peptide 1–42 in the eye. The parameters assessed included ommatidial organization, vacuole area, retinal thickness, and Aβ peptide quantification. The present study revealed that the fractions, particularly the peptidic fraction, significantly reduced the vacuole area and increased the retina thickness in treated flies, indicating an improvement in neurodegeneration phenotype. The peptidic fraction was also found to alter Aβ aggregation dynamics, inhibiting Aβ fibril formation, as revealed by dynamic light scattering. This study demonstrated that kefir fractions, particularly the peptidic fraction < 10 kDa, have the potential to regulate Aβ aggregation and alleviate neurodegeneration in a *Drosophila melanogaster* AD-like model. These findings suggest that kefir fractions could be viable for the bioprospection of novel drug prototypes for AD treatment, providing valuable insights into strategies targeting Aβ aggregation and neurodegeneration in AD.

**Supplementary Information:**

The online version contains supplementary material available at 10.1038/s41598-024-76601-9.

## Introduction

Alzheimer’s disease (AD) is known to be a progressive neurodegenerative pathology associated with aging^1^. Among the various hypotheses and mechanisms that explain this phenomenon, the amyloidogenic pathway hypothesis is one of the most recognized^2^. This pathway describes the processing of the amyloid-beta precursor protein (APP), which, when cleaved by the enzyme β-secretase (BACE) followed by γ-secretase, produces peptide fragments of 40 and 42 amino acids^3^ that accumulate extracellularly, contributing to the formation of so-called amyloid plaques^4,5^. This process is also associated with inflammatory processes^6^ and is responsible for the disruption of synapses and neuronal loss^7^.

In addition to amyloid plaque formation, tau aggregation plays a key role in the pathogenesis of Alzheimer’s disease^8^. Tau proteins regulate the function of microtubules in neurons and typically contain 2 to 3 moles of phosphates per mole of protein, but in AD brains they are found to be elevated^2^. When hyperphosphorylated, it can form neurofibrillary tangles in neurons, leading to dysfunction and cell death, and is correlated with cognitive decline in Alzheimer’s patients^2^.

Due to its high incidence in the population and its devastating effects on affected patients^9^, ways to mitigate these losses and symptoms have been constantly sought. One approach to better understand the underlying AD mechanisms and test potential drugs is the use of model organisms^10^. Several organisms can be used, including invertebrates, fish, and mammals^11–13^, but the advantages of *Drosophila melanogaster* are well known, ranging from ease of genetic manipulation and phenotypic inference^14–16^. The modeling of neurodegenerative diseases has been carried out using eye-directed expression drivers, which offer a range of possibilities for analysis related to neurodegeneration^17^. Considering that one of the main features of AD is the production and extracellular accumulation of amyloid beta (Aβ), the overexpression of these human peptides in fruit flies allows the study and screening of compounds^18^ that can alleviate the tissue damage, memory deficits, and other behavioral outcomes caused by Aβ.

Several compounds and their effects have been investigated in studies of AD to understand how they can be applied to the treatment of the disease^19–22^. When utilizing *D. melanogaster*, the first way to identify the therapeutic potential of a compound is based on its ability to modify the neurodegenerative phenotype presented in the model organism^16,23^, and in previous studies, kefir and its compounds have been shown to have this capacity^24,25^. Kefir has gained prominence in the literature due to its health benefits. As a probiotic, it may help modulate the intestinal microbiota, which directly impacts neurodegenerative and inflammatory processes through the gut-brain axis^26,27^. Studies in human and animal models have shown beneficial effects of kefir in several forms: *in natura*^28,29^, in its cell-free fraction^30,31^, in its metabolic^24^ and peptide fractions^25^ and its purified peptides^32,33^.

In our previous studies, Batista et al. (2021)^24^ treated flies with kefir in natura and metabolic fractions. The author also describes obtaining the metabolic fraction from filtrations and using organic solvents in a liquid-liquid partition to separate compounds of increasing polarity, as well as identifying these metabolites produced during fermentation and identifying the main microorganisms present, based on a metabolome analysis and 16 S sequencing. The author describes the presence of compounds with antioxidant, anti-inflammatory, and anti-BACE activity in all metabolic fractions which would explain the improvements seen in the AD-like characteristics evaluated.

Meanwhile, Malta et al. (2022)^25^ executed physical separation processes to obtain a fraction rich in peptides, which were confirmed by a proteopeptidomic analysis. In this work, identification of peptides sequences and in silico analysis were performed to predict the interaction of these peptides with the main targets present in the model used: BACE, APP and Acetylcholinesterase enzyme (AchE).

The fractions obtained in both works were tested and were able to reduce the AD-like phenotype in flies overexpressing both human BACE and APP^24,25^. Here, we investigate the effect of peptide and metabolic fractions on the neurodegenerative phenotype of *D. melanogaster* that overexpress the human Aβ 1–42 peptide in the eye. The effects were analyzed through morphological and quantitative indicators, demonstrating that these fractions may interact directly with Aβ 1–42 and can improve the AD-like phenotype.

## Results

### Model validation (scanning electron microscopy and quantitative analysis, histopathological analysis and relative amyloid beta quantification)

**Fig. 1:**
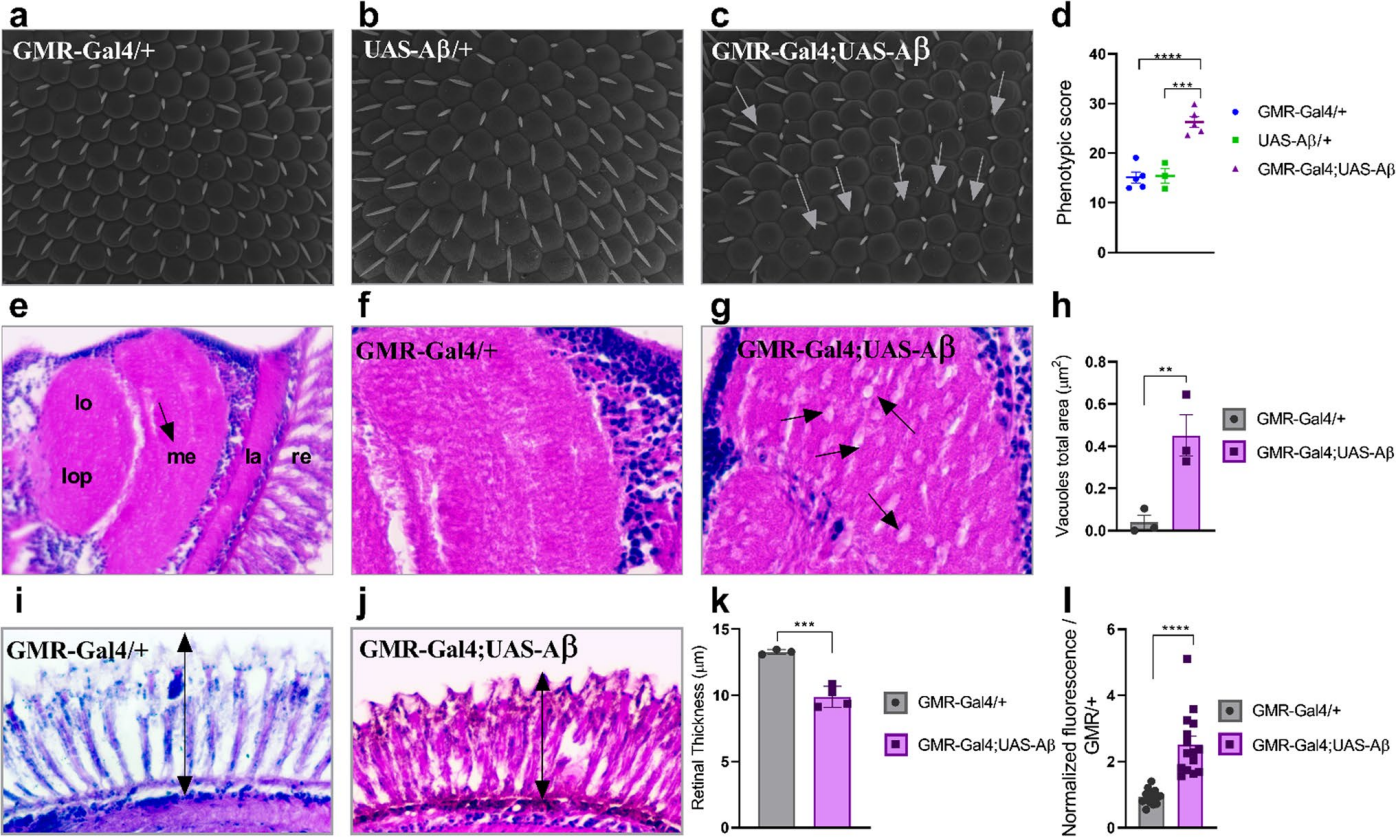
Flies with the genotype GMR-Gal4/+;UAS-Aβ/+ showed a degenerative phenotype. Representative SEM images obtained at 1200x magnification. (**a**) GMR-Gal4/+ (**b**) UAS-Aβ/+ (**c**) GMR-Gal4;UAS-Aβ; white arrows indicate disordered ommatidia (**d**) Phenotypic score determined through quantitative analyses using the Flynotyper plugin on ImageJ, flies GMR-Gal4;UAS-Aβ presented a higher score than both the GMR-Gal4/+ and UAS-Aβ/+ controls (*p* < 0.0001 and *p* = 0.0004, respectively)*n ≥ 3.***e**) Representative 3-µm paraffin sections of the fly head GMR-Gal4/+, 40× magnification indicating the different parts of the visual system in the optic lobe: retina (re), lamina (la), medulla (me), lobe (lo), and lobe plate (lop); the black arrow indicates the regions analyzed for vacuolar lesions. **f**) GMR-Gal4/+ at 100× magnification. **g**) GMR-Gal4/+;UAS-Aβ/+ at 100× magnification; the black arrows indicate vacuolar lesions. **h**) Total vacuole area. There was a greater area of vacuoles in the GMR-Gal4;UAS-Aβ fly than in the control genotype (*p* = 0.0083) *n* = 3. **i**) Representative retinal thickness of GMR-Gal4/+, 100× magnification; the black arrow indicates the measured region. **j**) Representative retinal thickness of GMR-Gal4/+;UAS-Aβ/+, 100× magnification; the black arrow indicates the measured region. **k**) Retinal thickness quantification. The GMR-Gal4;UAS-Aβ flies presented a thinner retina than the genotype control (*p* = 0.0009) *n* = 3. **l**) Relative amyloid content in the GMR-Gal4;UAS-Aβ flies presented a greater amyloid content than the GMR/+ flies (*p* < 0.0001) triplicate of pool with 10 heads in each. All data are shown as individual values, the mean ± S.E.M. (two-tailed ANOVA and unpaired *t* test) and all images are of flies at 1–2 days post-eclosion (d.p.e.).

First, the model was validated by comparing control (GMR-Gal4/+ and UAS-Aβ/+) and AD-like (GMR-Gal4/+;UAS-Aβ/+) genotypes. Morphological and quantitative analyses of the ocular surface (Fig. 1a-d), histopathological analyses of the medulla (Fig. 1e-h) and retina (Fig. 1i-k), and relative quantification of beta-amyloid (Fig. 1l) were performed to validate the model.

The qualitative analysis was performed using images obtained by scanning electron microscopy (SEM) at 1200x magnification. A uniform distribution pattern of ommatidia was observed in the controls (Fig. 1a-b), whereas this was not observed in the GMR-Gal4;UAS-Aβ genotype (Fig. 1c). To quantify the level of disorder, two hundred ommatidia per image were analyzed at 550× magnification, and the results revealed a significantly higher level of disorder in the AD-like model (GMR-Gal4;UAS-Aβ) than in the GMR-Gal4/+ (*p* < 0.0001) and UAS-Aβ/+ (*p* = 0.0004) controls (Fig. 1d). The score obtained by the control groups shows normal ommatidial organization, while the AD-like model has an equivalent to subtle rough, according to Iyer et al. (2016)^34^. This result showed that the overexpression of Aβ peptide has a negative effect on ommatidium formation and organization. Additionally, there was no significant difference between the negative controls, therefore subsequent analyses were conducted using only GMR-Gal4/+ for comparison.

The histopathological analysis of retina and medulla sections from flies at 1–2 days post-eclosion (d.p.e.) confirmed the results obtained with SEM. Vacuolar lesions and retinal integrity are considered markers for neurodegeneration tissue damage in models using GMR-Gal4 as a driver strain. Therefore, the total area of vacuoles in the medulla region (Fig. 1f-g) and the retinal thickness (Fig. 1i-j) were quantified. The GMR-Gal4;UAS-Aβ flies exhibited more severe tissue damage, represented by a larger total vacuole area than the control flies (*p* = 0.008) (Fig. 1h**)** and a decrease in retinal thickness (Fig. 1k) (*p* = 0.0009), potentially indicating a neurodegenerative phenotype.

Using the Thioflavin T (ThT), the relative levels of Aβ were further quantified in flies at 1–2 d.p.e. (Fig. 1l), and the results showed that the AD-like model flies exhibited a greater amyloid content compared to the control flies (*p* < 0.0001). Taken together, these results suggest that the overexpression of the Aβ peptide in *D. melanogaster* eyes promotes a neurodegenerative phenotype.

### Treatments

#### Toxicity assay (metabolite and peptidic fractions)

The toxicity of the fractions on the embryos and their effect on hatching rate were evaluated before parameters related to the neurodegeneration phenotype were studied. The groups (*n* = 100/ group) were divided into the untreated (receiving water), vehicle group (receiving Tween 80 at 0.01%) and embryos treated with each fraction (< 10 kDa, EtAOc, DCM, Hex and, ButOH). The average hatching rate was 79.94% and analysis performed using the Chi-Square test, showed no significant difference (*p* > 0.5) between the groups treated with the fractions and the untreated controls and the healthy model group (GMR-Gal4/+).

#### Effects of the peptidic fraction from kefir on AD-like flies

**Fig. 2:**
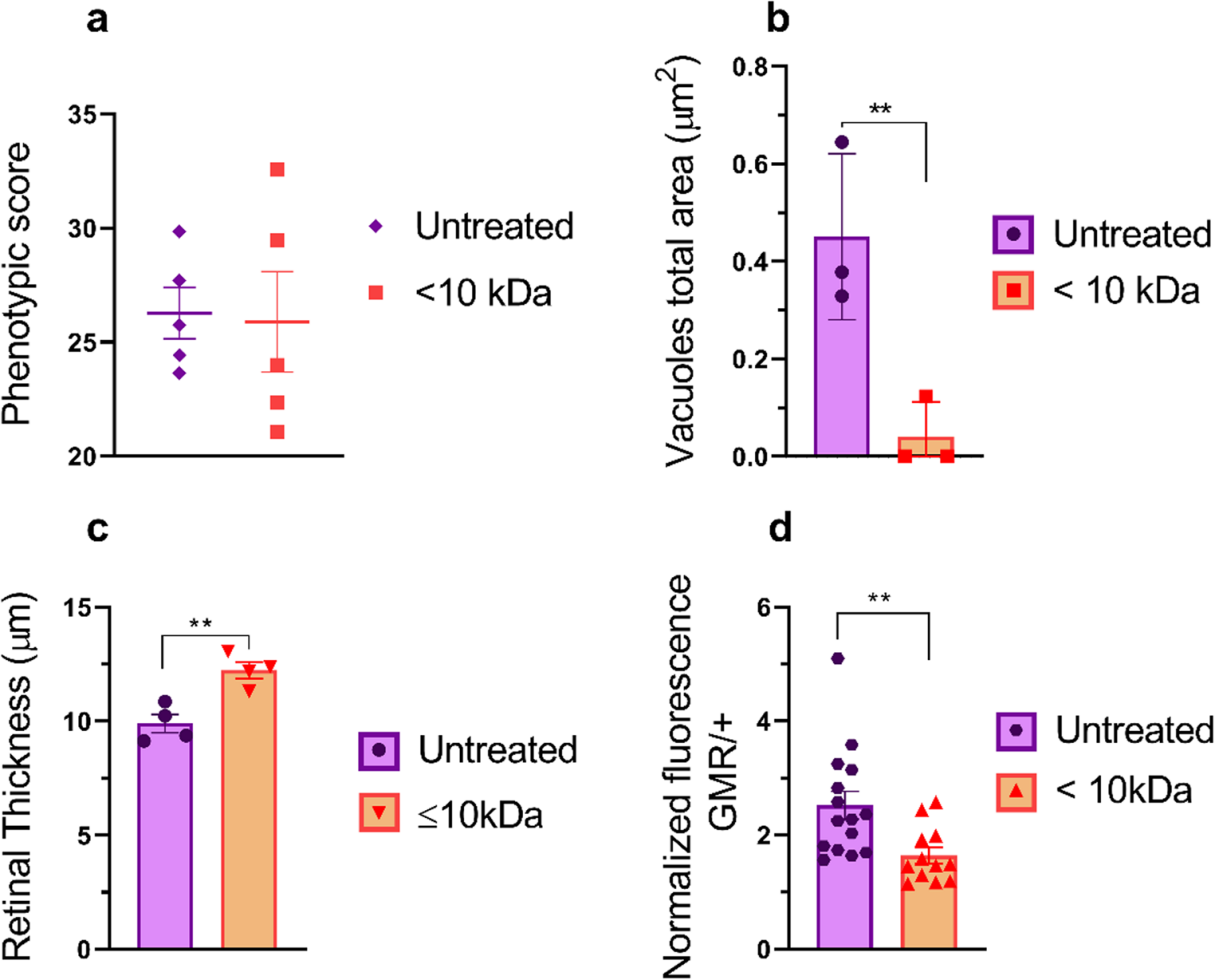
Quantitative analysis of GMR-Gal4/+; UAS-Aβ/+ flies treated with the peptidic fraction.(**a**) Phenotypic score determined through quantitative analyses using the Flynotyper plugin on ImageJ. Flies treated with the fraction < 10 kDa did not present any significant difference compared with untreated controls (*p* > 0.5) *n* = 5. (**b**) Total vacuole area. Flies treated with the < 10 kDa presented a significantly reduced total area of damage (*p* = 0.009) compared to control group *n* = 3. (**c**) Retinal thickness. Flies treated with the < 10 kDa fraction presented a greater retinal thickness (*p* = 0.005) than did control flies *n* = 3. (**d**) Relative amyloid quantification through a thioflavin assay. Flies treated with the < 10 kDa fraction presented a lower amyloid content than did untreated flies (*p* = 0.008) triplicate of pool with 10 heads in each. The data are shown as individual values, the mean ± S.E.M. (unpaired *t* test).

To investigate the effects of kefir peptidic fraction < 10 kDa treatment on the AD-like model (GMR-Gal4/+; UAS-Aβ/+), we initially evaluated the external structure of the eye (organization of ommatidia) in adult AD-model flies treated with and without peptide fraction. No significant differences were detected (*p* = 0.8799) (Fig. 2a).

However, the histological analysis of the group treated with the < 10 kDa fraction showed a significant decrease in the vacuole area (*p* = 0.009) compared to the untreated control (Fig. 2b). Additionally, a significant increase in retinal thickness (*p* = 0.005) was observed despite no difference in external morphology (Fig. 2c).

Treatment with kefir peptide fraction < 10 kDa also significantly decreased the amyloid content (*p* = 0.008) compared to that in the untreated control group (Fig. 2d**)**, indicating the potential of this fraction to interact with Aβ peptides. Representative images of flies for all analyses are shown in Supplementary Figure (S1).

#### Metabolic fractions in eye structure and integrity

**Fig. 3:**
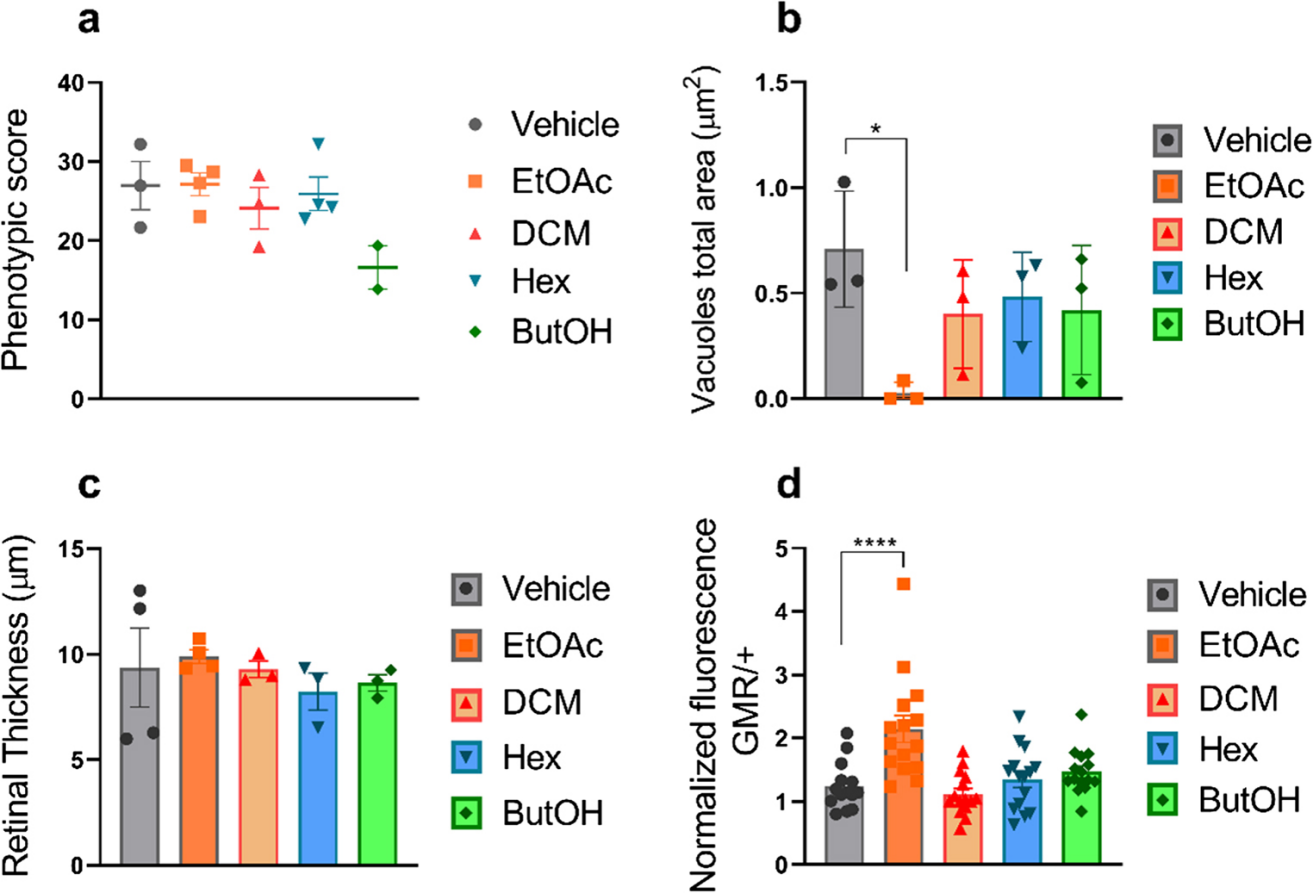
Quantitative analysis of metabolic fraction-treated flies. (**a**) Phenotypic score determined through quantitative analyses using the Flynotyper plugin on ImageJ. None of the treatment groups presented significant differences compared with the control group (vehicle) (*p* > 0.5) *n* ≥ 3. (**b**) Total vacuole area. Compared with control flies, only flies treated with the EtOAc fraction presented a reduced total area of vacuoles (*p* = 0.023) *n* = 3. (**c**) Retinal thickness. None of the treatment groups presented significant differences compared with the control group (vehicle) (*p* > 0.5) *n* = 3. (**d**) Relative amyloid quantification through ThT assay; only flies treated with the EtOAc fraction presented a greater amyloid content than did the control group (*p* < 0.0001) triplicate of of pool with 10 heads in each. The data are shown as individual values, the mean ± S.E.M. (unpaired *t* test).

Histopathological analyses were conducted to assess eye structure and integrity. Phenotypic comparisons of the external structure of the compound eye and the level of ommatidial organization showed no significant difference among the four groups treated with metabolic fractions (EtOAc, DCM, Hex and ButOH), compared to the control group treated with the vehicle. (*p* = 0.1010) (Fig. 3a).

Another characteristic evaluated was the total vacuole area. Compared to the vehicle group, only the group treated with the EtOAc fraction showed a significant reduction (*p* = 0.023) potentially indicating a higher integrity (Fig. 3b). Moreover, no significant differences in retinal thickness were found between the treated and untreated control groups (Fig. 3c) (*p* = 0.8415).

Relative Aβ quantification through thioflavin assay showed that only the group treated with the EtOAc fraction exhibited significantly greater relative levels of Aβ (*p* < 0.0001) compared to the untreated group (Fig. 3d).

Representative images of flies for all analyses are shown in Supplementary Figure (S1).

#### The < 10 kDa and EtAOc fractions alters the dynamic aggregation of Aβ peptides

**Fig. 4:**
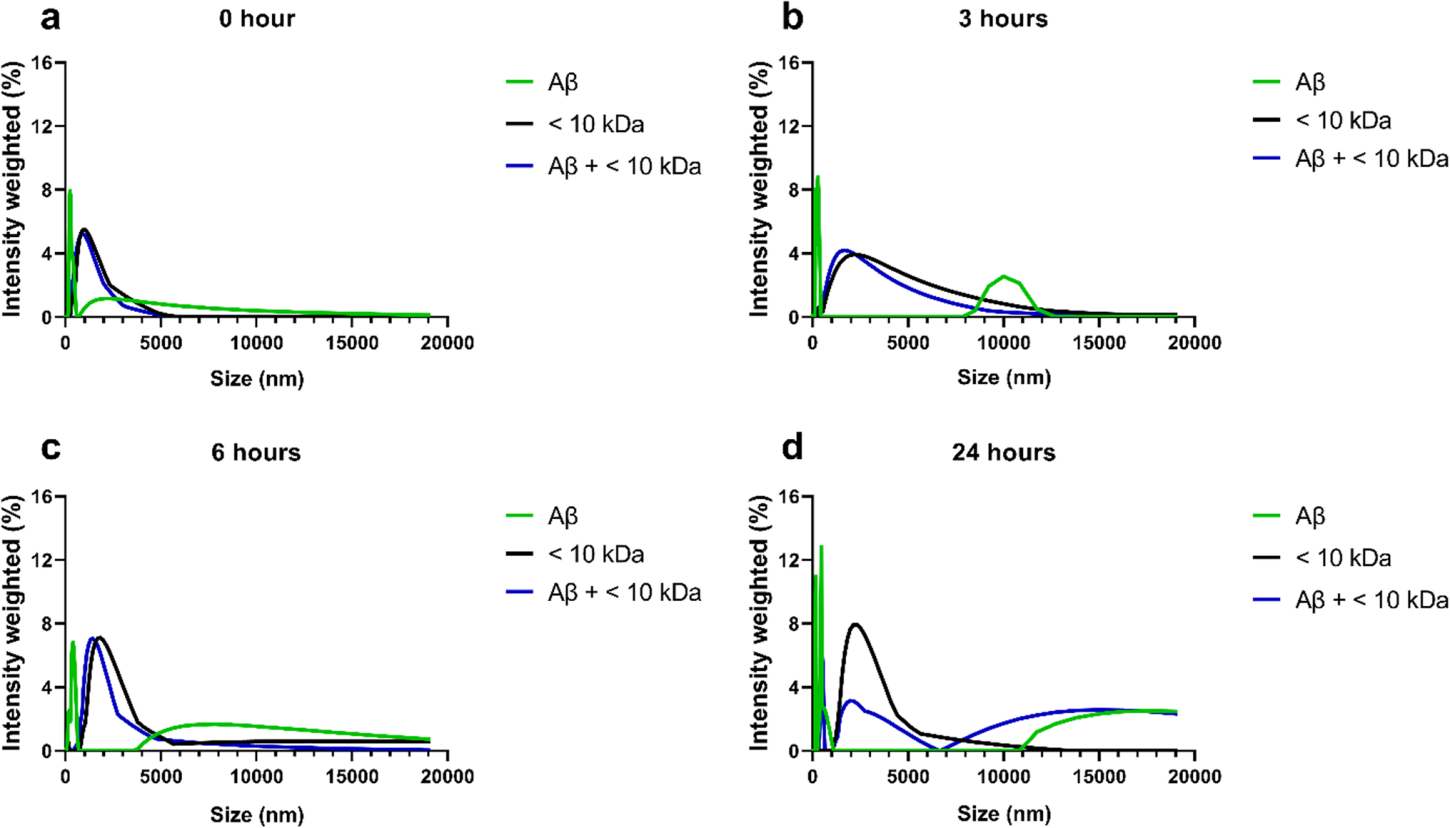
DLS measurements of Aβ aggregation. At the initial stage, minimal Aβ peptide aggregation is evident at (**a**) 0 h, with the appearance of initial oligomers at (**b**) 3 h, characterized by an intensity peak at 10,000 nm exclusive to the Aβ-only solution. This trend continued at approximately (**c**) 6 h, when Aβ aggregates started to form but remained absent in the Aβ + < 10 kDa solution. However, at (**d**) 24 h, a visible change occurs with the emergence of an intensity peak in the Aβ + < 10 kDa solution. This peak is shifted to the left compared to that of the Aβ-only solution, indicating smaller oligomer sizes despite the presence of aggregation.

The dynamics of Aβ peptide aggregate formation were analyzed at 0, 3, 6, and 24 h by analysis of the hydrodynamic radius using dynamic light scattering (DLS). At three hours, a peak corresponding to the formation of nanostructures in the 10,000 nm region was identified. By 6 h, nanostructures with sizes equal to or larger than 5,000 nm were observed, without a decrease in size up to the maximum size analyzed. At 24 h, the disappearance of nanostructures between 5,000 nm and 10,000 nm was noted, with the formation of nanostructures above 11,000 nm indicating the aggregation of Aβ fibrils (Fig. 4a-d).

When Aβ peptides were incubated with the < 10 kDa fraction, the compounds in this fraction modified the dynamics of Aβ aggregate formation. At 3 h, the peak in the 10,000 nm region was not observed in the spectra of the treated samples. At 24 h, the < 10 kDa fraction led to a reduction in the amount of Aβ nanostructures, starting from the 11,000 nm region (untreated) to the 7,000 nm region in the treated samples (Fig. 4a-d).

## Neurocytotoxicity of peptide fractions < 10 kDa and EtOAc

**Fig. 5:**
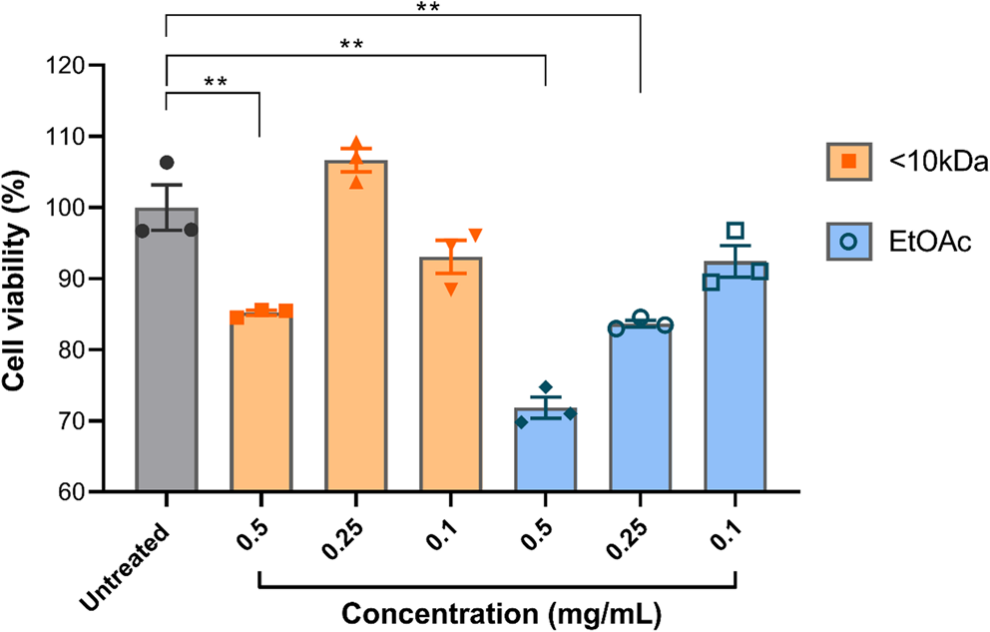
Cell viability of human neurons treated with EtOAc and <10kDa fractions. . Fraction <10 kDa at concentration of 0.5 mg/mL significantly reduces viability compared to the untreated group (*p* = 0.0099). The EtOAc fraction at a concentration of 0.5 and 0.25 mg/mL also significantly reduces the viability (*p* = 0.0013 and *p* = 0.0071 respectively). The data are shown as individual values, the mean ± S.E.M. (unpaired *t* test).

The effects of peptide fractions < 10 kDa and EtOAc on SHSY5Y cell viability were assessed. The peptide fraction < 10 kDa demonstrated significant neurotoxicity at the highest concentration tested 0.5 mg/mL (*p* = 0.0099), with no significant effects observed at concentrations 0.25 and 0.1 mg/mL in comparison with the untreated group (*p* = 0.1360 and *p* = 0.1548 respectively) (Fig. 5). In contrast, the EtOAc fraction significantly reduced cell viability at the two highest concentrations tested 0.5 and 0.25 mg/mL, (*p* = 0.0013 and *p* = 0.0071 respectively), whereas the lowest concentration (0.1 mg/mL) did not produce a significant effect in viability (*p* = 0.1232) compared to untreated group (Fig. 5).

### Preventive effect of kefir fractions < 10 kDa and EtOAc on Alzheimer’s disease-like culture cell model

**Fig. 6:**
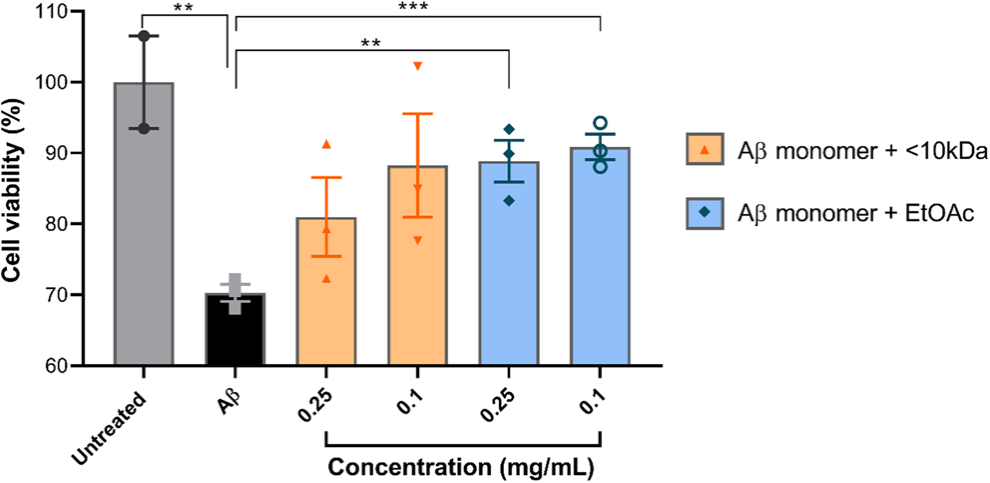
Cell viability of human neurons co-treated with EtOAc and < 10 kDa peptidic fraction from kefir and synthetic Aβ. Group that received Aβ significantly decreased the cell viability compared to the untreated group (*p*  = 0.0101). Only the treatments of synthetic peptide Aβ pre-incubated with EtOAc fraction at the concentration of 0.25 and 0.1 mg/mL showed an increased viability compared to the Aβ group (*p*  = 0.0044 and *p*  = 0.0007 respectively). The data are shown as individual values, the mean ± S.E.M. (unpaired *t* test).

As a control, an Aβ-only group compared to an untreated group showed a significant decrease in viability (*p* = 0.0101). The peptide fraction < 10 kDa showed no potential to inhibit Aβ peptide aggregation at either concentration tested (*p* > 0.5) (Fig. 6). However, the kefir fraction EtOAc showed inhibitory potential against Aβ peptide aggregation at both concentrations tested: 0.25 mg/mL treatment resulted in an 18% increase in viability (*p* = 0.0044) and 0.1 mg/mL resulted in a 20% increase in viability (*p* = 0.0007) compared to the cell group treated with Aβ alone (Fig. 6).

#### Effects of kefir fractions < 10 kDa and EtOAc on Alzheimer’s disease-like culture cell model (treatment)

**Fig. 7:**
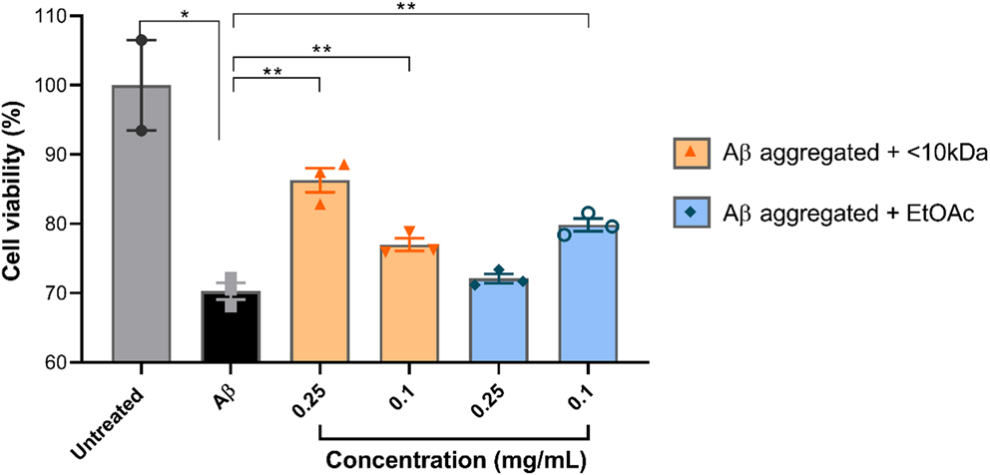
Cell viability of human neurons stimulated with synthetic Aβ aggregates then treated with EtOAc and < 10 kDa peptidic fraction from kefir. Cell incubated with Aβ aggregates for 48 h then treated with < 10 kDa fraction at concentrations of 0.25 and 0.1 mg/mL had an increased in viability (*p*  = 0.0017 and *p*  = 0.0116 respectively) compared to the Aβ group. Cells treated with EtOAc fraction at 0.1 mg/mL also had an increase in viability over the Aβ group (*p*  = 0.0033). The data are shown as individual values, the mean ± S.E.M. (unpaired * t* test).

The < 10 kDa peptide fraction showed the potential to reverse established senile plaques at both concentrations tested. Cells treated with the 0.25 mg/mL concentration increased cell viability by 15% (*p* = 0.0017) and those treated with the 0.1 mg/mL concentration increased cell viability by 7% (*p* = 0.0116), both compared to the control group treated with Aβ peptide alone. As for the treatment with the EtOAc fraction, cells treated at a concentration of 0.25 showed no significant difference compared to the control group (*p* = 0.2594), while treatment at a concentration of 0.1 mg/mL significantly increased cell viability compared to the control group treated with the Aβ peptide alone (*p* = 0.0033) (Fig. 7).

## Discussion

The beneficial health effects promoted by kefir have been demonstrated in the literature across various applications due to its biological activities^35,36^. Kefir’s use ranges from treating and recovering from dysbiosis on its own^37^ to serving as an adjuvant in the treatment of related diseases, including inflammatory^38^, neurodegenerative^39^ and metabolic syndrome^40^. The benefits of kefir have been demonstrated in both animal models^41^ and humans^42–47^. In previous works, our group demonstrated the positive effects of metabolic^24^ and peptide^25^ fractions in Alzheimer’s model flies overexpressing both human BACE and APP. In the present study, these findings were investigated in a *Drosophila melanogaster* model in which only Aβ 1–42 was expressed.

Only the fractions and concentrations that showed the best results for the parameters evaluated in our previous works were selected. As summarized by Batista et al.. 2021^24^, the chosen metabolic fractions at the selected concentrations had the highest capacity to reduce neurodegeneration indices, while Malta et al.. 2022^25^ identified peptide sequences and predicted their interaction with β-secretase, Aβ peptide, and acetylcholinesterase. Considering these possible interactions, a *Drosophila melanogaster* model with overexpression of the Aβ 1–42 peptide was chosen to validate these fractions against exclusively the human Aβ peptide expressed in this model, as the strain used lacks β-secretase, reducing potential interaction targets.

Alzheimer’s disease has been extensively studied in *D. melanogaster* model^15,48–50^. By using an eye driver (GMR-Gal4) to express the human Aβ 1–42 peptide, a complex yet accessible model can be created^34^. The model is easy to handle and phenotype, allowing for precise quantitative analysis of phenotypic parameters associated with neurodegeneration processes^51–53^.

First, the model was validated by crossing flies containing the UAS-Aβ 1–42 peptide with an eye driver (GMR-Gal4). Other studies have also demonstrated similar alterations in neurodegeneration using an eye driver^18,54–56^, but relating our data to the findings in the literature was challenging due to the large number of drivers and responders inserted in different positions in the genome, resulting in different phenotypes^57^.

The peptidic fraction < 10 kDa improved all evaluated parameters except the organizational level of ommatidia, confirming the *in silico* prediction of Malta et al.. (2022)^25^ that showed peptides in this fraction can interact with Aβ plaques. As previously shown, this fraction contains many peptides that can interact with more than one element of the amyloidogenic pathway. The fly model analyzed in this work infers that the interaction between the fraction and Aβ peptides occurs and can alter the phenotype.

According to Batista et al.. (2021)^24^, in the APP-BACE expression model in the brain, all metabolic kefir’s fractions decrease the neurodegeneration index (vacuole area). In this present study, only the EtOAc fraction reduced the total area of vacuoles. The lack of similarity in results between both studies may be due to the difference in the fly models used^57^. This result shows that only the EtOAc can interact with beta-amyloid peptides, while the other ones probably could inhibit the activity of beta-secretase enzyme or interact with the APP.

The DLS measurements underscore the inhibitory effect of the < 10 kDa fraction on Aβ aggregation, confirming our previous in silico findings^25^. In this work, we provide the first experimental evidence that the < 10 kDa fraction can interact with Aβ plaques to promote dynamic aggregation changes. The DLS has been used in studies of dynamic aggregation (or anti-aggregation) of Aβ^58,59^. However, additional pharmacological investigations are necessary to confirm this inference.

As a proof of concept for the dynamic anti-aggregation effects of synthetic Aβ peptide by kefir fractions, a human neuron cell culture Alzheimer’s-like model was employed. For preventive purposes, kefir fractions were incubated with synthetic Aβ peptide monomers. In the treatment evaluation, synthetic Aβ peptide monomers were first allowed to aggregate over 48 h, followed by their introduction to the neuron culture and a subsequent 48-hour incubation to induce senile plaque formation. Post plaque formation, kefir fractions were administered to assess their potential in mitigating plaque-induced toxicity. The results from the neuron cell culture experiments agree with the dynamic light scattering (DLS) analysis and align with our previous findings in a *D. melanogaster* model^25^.

To date, our research group has conducted in silico, in vitro, and in *vivo* studies, with the latter limited to invertebrate models. For the first time, we have extended our analyses to a human neuron culture model. While the results suggest positive effects of the kefir fractions tested, further research is warranted, especially in a murine model of Alzheimer’s disease. It is also notable that, although DLS provided valuable insights into particle size, aggregation, and sample homogeneity, it did not identify which species of Aβ42 kefir fractions stabilizes. A more detailed characterization of these structures could provide valuable insights and should be addressed in future research. Furthermore, it would be beneficial to investigate the biological pathways associated with Aβ42 through techniques such as Western blot and immunostaining. This could elucidate whether kefir peptides interact directly with Aβ42 or influence distinct pathways.

In conclusion, the outcomes of this study are consistent with previous findings reported by our research group, further substantiating the interaction between peptides and amyloid beta. The evaluated kefir fractions represent prospective candidates for the development of prototypes aimed at modulating amyloidogenic processes in drug discovery.

## Methods

### Drosophila stock

The Drosophila strains used in this study included *w*^*1118*^ (BL#3605), UAS-Aβ (UAS-A.beta1-42 BL#64216), and GMR-Gal4 (GAL4-ninaE. GMR BL#1104) strains obtained from the Bloomington Drosophila Stock Center. The flies were maintained on Bloomington standard culture media at 25 °C under a 12:12 h light/dark cycle during the expansion period.

To obtain flies with the desired genotype, virgin females from the UAS-Aβ strain were crossed with GMR-Gal4 males. The pairs were placed in an egg-laying medium, and the embryos were collected after 8 h of oviposition. Parental controls were obtained from the crosses GMR-Gal4 with *w*^*1118*^ as well as with UAS-Aβ x *w*^*1118*^ following the same procedures.

### Treatment

For the treatment, kefir fractions were used with the same sample previously isolated as described by Malta et al. 2022^25^ and Batista et al. 2021^24^. The concentrations used were based on the best results of our previous work and are: kefir peptide fraction < 10 kDa at a concentration of 0.25 mg/mL and kefir metabolic fractions at the following concentrations: Hex (hexane) 0.1 mg/mL, DCM (dichloromethane) 0.25 mg/mL, EtOAc (ethyl acetate) 0.25 mg/mL, and ButOH (N-butanol) 0.25 mg/mL. After collection, the embryos were placed in vials containing 1 g of enriched mashed potato medium and hydrated with 5 mL of treatment solution, along with a group receiving the vehicle (Tween 80 0.01%) and untreated control (received medium with water only). After 48 and 72 h, 100 µL of treatment solution was added to the medium surface. Upon eclosion, flies aged 0–1 d.p.e. (days post eclosion) were collected, phenotypically separated, and maintained in untreated media for 24 h for subsequent analyses.

### Eclosion assay

The eclosion assay was adapted from Rand et al.. 2014^60^. Approximately 100 embryos from the performed crosses were placed in a medium containing treatment solution, along with an untreated control group, and 48 and 72 h later, 100 µL of treatment solution was added to the medium surface. After 12 days, the number of emerged adults in each vial was counted, and the eclosion percentage was calculated.

### SEM – Scanning electron microscopy

Flies at 1–2 d.p.e. from all treatments and the control were euthanized, dehydrated in absolute ethanol for 48 h, and then subjected to the critical point drying process. The samples were mounted on metal stubs covered with carbon-conductive tape and coated with gold. Images were acquired at magnifications of 300, 550, and 1200x using a Tescan VEGA 3 LMU electron microscope. The obtained images were analyzed using the Flynotyper plugin, available at flynotyper.sourceforge.net, and integrated with ImageJ software for the quantification of the organizational level of ommatidia, represented as a phenotypic score that ranges from 0 to 100, with higher scores indicating more severe disorganization and damage.

### Histopathological analysis

For histological analysis, five adult flies of the GMR-Gal4;UAS-Aβ strain 1-2 days post eclosion were collected from each control/treatment group, anesthetized with ethyl ether and fixed in Carnoy solution (6: 3: 1, 99% ethanol, chloroform and glacial acetic acid) for 24 h and processed in 70% ethyl alcohol (2x), 80% ethyl alcohol (2x), 90% ethyl alcohol (2x), absolute ethyl alcohol (2x), and xylol (2x) for 15 min in each repetition and 60% liquid paraffin (2x) for 30 min. The fly heads were embedded in paraffin, and the blocks were sectioned at 3 μm thickness using a semiautomatic microtome (SLEE CUT5062).

The sections were hydrated, stained with hematoxylin and eosin, mounted and

photographed with a light photomicroscope. Medulla (optic lobe) of three or more adult flies was used to calculate the neurodegeneration index and area of vacuolar lesions using the ImageJ software.

### Relative quantification of Aβ using ThT

To confirm the amyloidogenic pathway in AD-like model, relative amyloid levels were measured using Thioflavin T (ThT), a benzothiazole dye that shows increased fluorescence when it binds to amyloid fibrils^61^. The relative quantification of Aβ using ThT was previously standardized in^25,62^. Flies aged 1–2 d.p.e. were collected from all treatments and controls, euthanized in liquid nitrogen, and stored at -80 °C until the next steps. On the day of the experiment, the heads were collected and homogenized in 1X PBS, and this entire process was carried out on ice. The homogenate was then centrifuged for 2 min at 10,000 *× g* at 4 °C, and the supernatant was collected and used for total protein quantification by the Bradford method; amyloid quantification was performed using thioflavin T (ThT). A 2 µL sample was incubated in a 96-well black plate with 198 µL of 10 µM ThT filtered solution for 20 min under agitation. Fluorescence was measured at 450 nm excitation and 482 nm emission and normalized in several steps. First, the autofluorescence of ThT in the absence of protein homogenate was subtracted as background. Then, the total fluorescence was adjusted according to the protein content of each sample (µg). Finally, the fluorescence values were normalized to the GMR-Gal4/+ control group, which does not express Aβ42, by dividing the fluorescence of each sample by the average fluorescence of the control group. For this assay, 3 pools of 10 heads for each group were made and a quintuplicate technique was performed.

### Synthetic Aβ preparation

The Aβ peptide was synthesized by AminoTech (Brazil) with a purity of 95%. Following the protocol described by Ryan et al. (2013)^58^, the synthetic peptide was first dissolved in 10% ammonium hydroxide (w/v) at a concentration of 0.5 mg/mL. It was then incubated at room temperature for 10 min, sonicated for 5 min, aliquoted, and lyophilized. The lyophilized peptide was reconstituted in 60 mM NaOH, resulting in a stock solution with a final concentration of 886 µM, and stored at -20 °C.

### Dynamic light-scattering (DLS) measurement

The size of the Aβ aggregates generated in the presence or absence of the < 10 kDa was measured using a dynamic light scattering instrument (LitesizerTM 500, Anton Paar).

All measurements were conducted at 25 °C with a detection angle of 90°. For this purpose, 1 µM Aβ 1–42 in 1× PBS was incubated either alone or with 0.25 mg/mL of the < 10 kDa fraction, and a solution containing only the < 10 kDa fraction at 0.25 mg/mL was used as a control. All samples were filtered through a 0.22 μm Millipore filter.

These three preparations were analyzed for their particle size at different time points (0, 3, 6, and 24 h). For the readings, 2 mL of each sample was used, and the samples were kept at rest between measurements. The intensity of the size distribution was obtained through analysis in Kalliope software.

### Human cell culture

The cell line of human neuroblastoma was used, SH-SY5Y, donation from University of Siena, Italy, was maintained at 37 °C in a humidified atmosphere, with 5% CO2. Cells were cultured in Dulbecco’s modified Eagle’s medium (DMEM, Cultilab^®^) supplemented with 10% fetal bovine serum (FBS) (Cultilab^®^) and 1% antibiotic (Vitrocell^®^).

### Treatment

Kefir fractions < 10 kDa and ethyl acetate (EtOAc) were diluted in 3 different concentrations (0.5, 0.25, 0.1 mg/mL) and filtered through a 0.22 μm Millipore filter.

### Human neurotoxicity

Cells were plated in a 96-well plate at 10,000 cells per well in 100 µL DMEM and kept in an incubator for 24 h for adherence. At the end of this period, the treatments (kefir fractions) were added to the cells and each group was treated at one concentration. Controls consist of untreated cells as a negative control group and positive controls as treatment with 0.2% Triton-x.

At the end of the treatment period, cells were evaluated for cell viability using the AlamarBlue assay (Invitrogen^®^) by incubation with 10% (10 µl) resazurin at 37 °C for approximately 3 h. Resazurin is a non-fluorescent blue dye that can be reduced intracellularly to resorufin, which has a highly fluorescent pink color and is detected by colorimetric reading (absorbance at 570 and 600 nm).

### Cell culture Alzheimer-like model (preventive)

The synthetic Aβ peptide (stock solution 886 µM) was first diluted to a final concentration of 1 µM and then filtered through a 0.22 μm Millipore filter. To evaluate the efficacy of the treatment in inhibiting Aβ peptide aggregation, the kefir fractions were mixed with the Aβ peptide and incubated at 37 °C for 48 h. Cells were seeded in a 96-well plate at a density of 5,000 cells per well, supplemented with 100 µL DMEM/well and incubated for 24 h to allow for cell attachment. Subsequently, Aβ peptide and kefir fractions were applied to the cells, and incubated for an additional 48 h, resulting in a total incubation time of 96 h. To ensure accurate comparisons, a positive control group consisting of untreated cells, a negative control group consisting of dead cells in DMEM with 0.2% Triton-X, and a control group of untreated Aβ peptide were included.

### Cell culture Alzheimer-like model (treatment)

Amyloid-beta peptide (886 µM) was diluted to a final concentration of 1 µM, filtered through a 0.22 μm membrane for sterilization, and incubated at 37 °C for 48 h to induce peptide aggregation. Cells were seeded in a 96-well plate at a density of 5,000 cells per well in 100 µL DMEM and incubated for 24 h. After incubation, Aβ peptide was then added to the cell culture, followed by an additional 48-hour incubation to facilitate the formation of senile plaques.

After this second incubation period, the cells were treated with kefir fractions at the most effective concentrations, and the incubation was extended for an additional 24 h to assess the potential of these treatments to reverse pre-established senile plaques.

### Statistical analysis

Data analysis was performed using GraphPad Prism 10. The normality of the data was assessed using the D’Agostino and Pearson test. For normally distributed data, groups were compared using a t-test, and for comparisons involving more than two groups, one-way ANOVA followed by Tukey’s multiple comparison test was used. For data that did not follow a normal distribution, non-parametric tests such as the Mann-Whitney U test (for two groups) or Kruskal-Wallis test followed by Dunn’s multiple comparison test (for more than two groups) were applied. A significance level of *p* < 0.05 was established for all tests.

## Supplementary Information

Supplementary Material 1.

## Data Availability

The datasets generated during and/or analyzed during the current study are available from the corresponding author on reasonable request.
