# Supplementary material for: Brazilian kefir fraction mitigates the Alzheimer-like phenotype in Drosophila melanogaster with β-amyloid overexpression model
Source: Sci Rep. 2024 Oct 26;14:25474. doi: 10.1038/s41598-024-76601-9 (PMC11513133; doi:10.1038/s41598-024-76601-9)
Supplement: Supplementary file 1 — Supplementary Material 1. [file 41598_2024_76601_MOESM1_ESM.pdf]

Supplementary Material

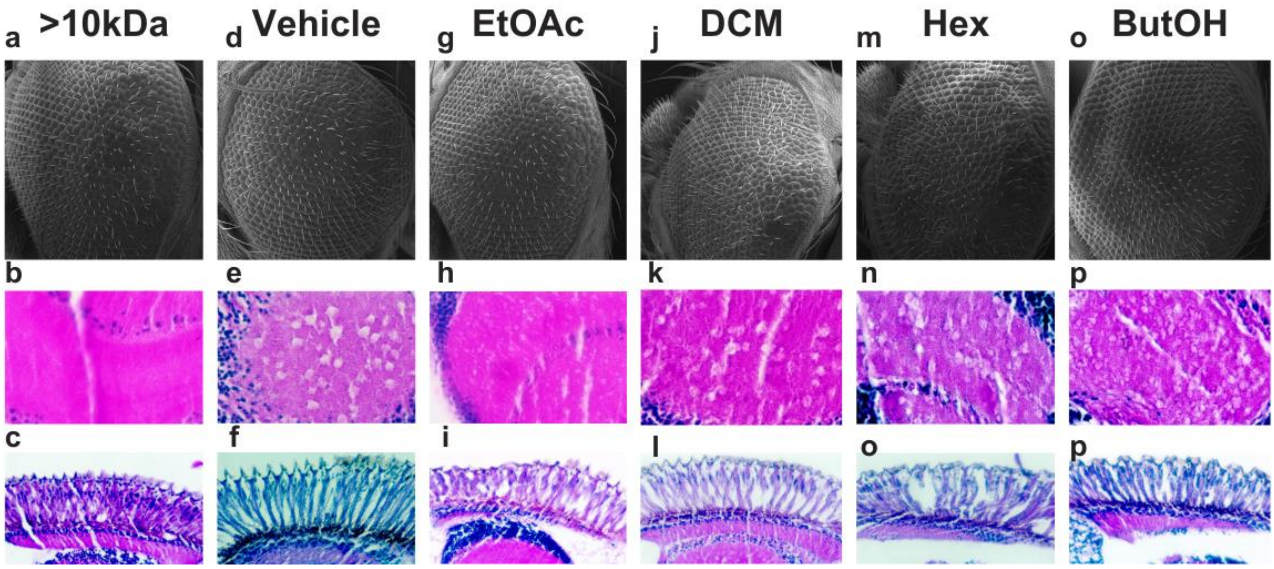

S1: Effects of kefir fractions and solvents in the Drosophila melanogaster Alzheimer's model (Aβ42) on the morphologies of ommatidia, medulla, and retina. The representative images show: (a, d, g, j, m, o) scanning electron micrographs of the compound eye; (b, e, h, k, n, p) histologic sections of the medulla; and (c, f, i, l, o, p) histological sections of the retina of Drosophila melanogaster treated with the different fractions (>10 kDa, vehicle, ethyl acetate [EtOAc], dichloromethane [DCM], hexane [Hex] and butanol [ButOH]) 1-*2* days post eclosion.
